# Trends in lifetime risk and years of potential life lost from diabetes in the United States, 1997–2018

**DOI:** 10.1371/journal.pone.0268805

**Published:** 2022-05-24

**Authors:** Alain K. Koyama, Yiling J. Cheng, Ralph Brinks, Hui Xie, Edward W. Gregg, Annika Hoyer, Meda E. Pavkov, Giuseppina Imperatore

**Affiliations:** 1 Division of Diabetes Translation, National Center for Chronic Disease Prevention and Health Promotion, Centers for Disease Control and Prevention, Atlanta, GA, United States of America; 2 Institute for Biometry and Epidemiology, German Diabetes Center, Düsseldorf, Germany; 3 Medical Biometry and Epidemiology, Faculty of Health, Witten/Herdecke University, Witten, Germany; 4 Department of Epidemiology and Biostatistics, School of Public Health, Imperial College London, United Kingdom; 5 Biostatistics and Medical Biometry, Medical School OWL, Bielefeld University, Bielefeld, Germany; Dr Baba Saheb Ambedkar Medical College and Hospital, INDIA

## Abstract

**Background:**

Both incidence and mortality of diagnosed diabetes have decreased over the past decade. However, the impact of these changes on key metrics of diabetes burden–lifetime risk (LR), years of potential life lost (YPLL), and years spent with diabetes–is unknown.

**Methods:**

We used data from 653,811 adults aged ≥18 years from the National Health Interview Survey, a cross-sectional sample of the civilian non-institutionalized population in the United States. LR, YPLL, and years spent with diabetes were estimated from age 18 to 84 by survey period (1997–1999, 2000–2004, 2005–2009, 2010–2014, 2015–2018). The age-specific incidence of diagnosed diabetes and mortality were estimated using Poisson regression. A multistate difference equation accounting for competing risks was used to model each metric.

**Results:**

LR and years spent with diabetes initially increased then decreased over the most recent time periods. LR for adults at age 20 increased from 31.7% (95% CI: 31.2–32.1%) in 1997–1999 to 40.7% (40.2–41.1%) in 2005–2009, then decreased to 32.8% (32.4–33.2%) in 2015–2018. Both LR and years spent with diabetes were markedly higher among adults of non-Hispanic Black, Hispanic, and other races compared to non-Hispanic Whites. YPLL significantly decreased over the study period, with the estimated YPLL due to diabetes for an adult aged 20 decreasing from 8.9 (8.7–9.1) in 1997–1999 to 6.2 (6.1–6.4) in 2015–2018 (p = 0.02).

**Conclusion:**

In the United States, diabetes burden is declining, but disparities by race/ethnicity remain. LR remains high with approximately one-third of adults estimated to develop diabetes during their lifetime.

## Introduction

The prevalence of diagnosed diabetes among adults in the United States is estimated at 34.1 million individuals in 2018, with 1.5 million newly diagnosed individuals per year, creating a substantial health and economic burden [1]. While metrics such as prevalence and incidence provide essential information regarding the descriptive epidemiology of diabetes, the lifetime risk (LR) of developing diabetes and years of potential life lost (YPLL) to diabetes provide additional crucial and practical information regarding diabetes burden for policy-makers, clinicians, researchers, and the general population. The LR, a cumulative incidence function, represents the probability of incident diabetes over one’s remaining lifespan, which is independent from the age distribution of the population and accounts for competing risks. The YPLL incorporates age at death, and therefore places more emphasis on premature death at younger ages compared to crude mortality rates [2]. Another related measure, the years spent with diabetes, measures the estimated number of years lived with diabetes for an individual in the general population. Unlike a point prevalence, this measure is not contingent on diabetes status, as it represents a measure of disease burden for an average individual in the entire population.

Previous studies in high income countries have estimated that over one third of individuals would develop diabetes during their lifetime [3–5]. In the United States, after an increase of diabetes incidence over two decades, diabetes incidence, as well as mortality among individuals with and without diabetes, have decreased [6, 7]. However, more current reports have not examined the impact of these changes on LR, YPLL, and years spent with diabetes, which are affected by incidence of diabetes and mortality among both individuals with and without diabetes. In addition, differences in incidence and diabetes outcomes by sex and race/ethnicity persist, but it is unknown how these disparities may also be reflected in the LR, YPLL, and years spent with diabetes. The objectives of this study were therefore to: 1) describe the trends in LR, YPLL, and years spent with diabetes in the general adult population of United States; and 2) evaluate any differences in these estimates by sex and race/ethnicity using a dynamic multistate model.

## Methods

### Study sample

Data from the 1997 to 2018 National Health Interview Survey (NHIS) were used to estimate the prevalence and incidence of diabetes. The NHIS is an annual multistage complex probability survey conducted by the National Center for Health Statistics (NCHS) of the Centers for Disease Control and Prevention (CDC) [8]. The cross-sectional household survey uses interviewer-administered questionnaires to assess the health status of eligible adults which include the civilian noninstitutionalized population residing in the United States. The survey is administered using a computer-assisted personal interviewing mode to ensure accurate data collection. Data on all-cause mortality through December 31, 2015 was obtained using the public use linked mortality file which utilizes probabilistic record matching with the National Death Index (NDI) [9]. Adults were excluded if they were not eligible for linkage due to insufficient identifying information needed to conduct the linkage [9]. A flow diagram showing the derivation of the analytic samples used is shown in **S1 Fig**. The Research Ethics Review Board of CDC approved the NHIS procedures and protocols, and written informed consent was obtained from all adults. Deidentified participant data and corresponding documentation are publicly available online at https://www.cdc.gov/nchs/nhis/1997-2018.htm [8].

### Diabetes status

Self-reported diagnosed diabetes was used to identify individuals with diabetes if they responded yes to the question: “Have you ever been told by a doctor or other health professional that you have diabetes or sugar diabetes (other than during pregnancy for female adults)?” Individuals with diabetes were also asked their age of diagnosis. The duration of diabetes was calculated as years from the age at diagnosis to the age at interview. Incident diabetes was defined as having self-reported diagnosed diabetes with a duration of one year or less. To account for individuals with a birthday within one year of incident diabetes, we assumed half of individuals with a diabetes duration of one year had incident diabetes within the past year [10]. Adults aged 18 to 84 who were not pregnant were included for the current study.

### Demographics

Demographic characteristics included self-reported age at interview (years), sex (male, female), education (less than high school, high school graduate or equivalent, more than high school), and race/ethnicity (non-Hispanic White, non-Hispanic Black, Hispanic, and other). Adults who had more than one race/ethnicity selected the single group they felt best described them.

### Statistical analysis

All analyses accounted for the complex sampling design to produce correct variance estimates using the Taylor series linearization method. For the mortality analysis, linkage eligibility adjusted weights were used. For calculating the average yearly incidence of diabetes, we aggregated the survey data into five periods: 1997–1999, 2000–2004, 2005–2009, 2010–2014, and 2015–2018. Except for sample sizes in Table 1, all analyses and resulting data are weighted to produce population-based nationally representative estimates of the United States. Logistic regression was used to model the prevalence of diabetes. Poisson regression was used to model incidence and all-cause mortality by prevalent diabetes. Discrete Poisson regression was used to estimate mortality by diabetes status; age at interview and time period were treated as time-dependent variables [11]. The log of person-time was used as an offset term to account for varying follow-up time. Regression models included age, age squared, sex, and race/ethnicity as covariates and the interaction of each single order term. Adults did not have any missing data for these variables. Mortality rates for adults in the 2000 to 2014 survey cycles were directly estimated. Because there was a limited number of deaths during the shorter first time period (1997–1999) and at the time of this analysis mortality data were not yet available from NCHS for the 2016 to 2018 annual surveys, we used Poisson regression with the time period as a continuous variable to linearly predict the mortality rates for these two periods. Regardless of their statistical significance, interaction terms between time period, age, sex, and race/ethnicity were included in the model to differentiate marginal estimates by time period and demographic group. Marginal averages were used to predict crude and adjusted prevalence, incidence, and mortality by demographic characteristics.

**Table 1:** Participant characteristics, prevalence and incidence of diagnosed diabetes, and mortality among aged 18 years and over^a^.

	Total *n* = 653,811	1997–1999 *n* = 97,231	2000–2004 *n* = 155,413	2005–2009 *n* = 125,343	2010–2014 *n* = 161,083	2015–2018 *n* = 114,741
Age, mean, years	45.3 (45.2–45.4)	44.1 (43.9–44.3)	44.5 (44.4–44.7)	45.1 (44.9–45.3)	45.8 (45.6–46.0)	46.5 (46.3–46.7)
Sex, %						
Male	48.4 (48.2–48.6)	48.2 (47.8–48.5)	48.2 (47.9–48.5)	48.5 (48.2–48.9)	48.5 (48.2–48.9)	48.5 (48.1–48.9)
Female	51.6 (51.4–51.8)	51.8 (51.5–52.2)	51.8 (51.5–52.1)	51.5 (51.1–51.8)	51.5 (51.1–51.8)	51.5 (51.1–51.9)
Race/ethnicity, %						
Non-Hispanic White	69.2 (68.7–69.6)	74.7 (74.0–75.3)	72.7 (72.0–73.3)	69.4 (68.7–70.0)	67.0 (66.4–67.7)	64.3 (63.2–65.5)
Non-Hispanic Black	11.8 (11.5–12.1)	11.2 (10.8–11.7)	11.4 (10.9–11.9)	11.8 (11.3–12.2)	12.0 (11.6–12.5)	12.3 (11.7–13.0)
Hispanic	13.5 (13.2–13.9)	10.2 (9.7–10.7)	11.6 (11.1–12.0)	13.5 (13.0–14.0)	14.9 (14.4–15.4)	16.1 (15.1–17.1)
Other	5.5 (5.3–5.7)	3.9 (3.7–4.2)	4.4 (4.1–4.6)	5.4 (5.1–5.6)	6.1 (5.8–6.3)	7.2 (6.7–7.7)
Education, %						
Less than high school	15.5 (15.3–15.8)	18.8 (18.4–19.3)	17.7 (17.3–18.1)	16.2 (15.8–16.7)	14.1 (13.7–14.5)	12.2 (11.7–12.6)
High school	27.4 (27.2–27.7)	30.2 (29.7–30.7)	29.4 (29.0–29.8)	28.3 (27.9–28.7)	26.1 (25.7–26.5)	24.3 (23.9–24.8)
More than high school	57.0 (56.7–57.4)	51.0 (50.3–51.6)	52.9 (52.3–53.5)	55.5 (54.9–56.0)	59.8 (59.2–60.4)	63.5 (62.9–64.2)
Crude prevalence of diabetes, %	7.8 (7.7–7.9)	5.2 (5.0–5.4)	6.4 (6.3–6.6)	8.0 (7.7–8.2)	9.0 (8.8–9.2)	9.4 (9.2–9.7)
Crude incidence of diabetes, %	0.7 (0.6–0.7)	0.5 (0.5–0.6)	0.7 (0.6–0.7)	0.8 (0.8–0.9)	0.7 (0.7–0.8)	0.7 (0.6–0.7)
Crude mortality rate, per 1000 person-years						
With diabetes	31.6 (30.8–32.4)	43.8 (41.9–45.6)^c^	36.4 (35.1–37.8)	28.7 (27.4–30.1)	23.8 (22.1–25.4)	19.2 (17.8–20.6)^c^
Without diabetes	8.1 (8.0–8.3)	10.4 (10.1–10.6)^c^	9.1 (8.9–9.2)	7.7 (7.5–7.9)	6.6 (6.3–6.9)	5.7 (5.5–6.0)^c^
Adjusted mortality rate, per 1000 person-years^b^						
With diabetes	17.5 (17.1–18.0)	27.3 (26.1–28.4)^c^	20.9 (20.2–21.7)	15.9 (15.1–16.6)	11.7 (10.9–12.5)	9.0 (8.4–9.7)^c^
Without diabetes	10.0 (9.8–10.1)	14.4 (14.1–14.7)^c^	11.7 (11.5–11.9)	9.2 (8.9–9.4)	7.2 (6.9–7.6)	5.8 (5.6–6.1)^c^
Rate ratio (with diabetes vs. without diabetes)	1.8 (1.7–1.8)	1.9 (1.8–2.0)^c^	1.8 (1.7–1.9)	1.7 (1.6–1.8)	1.6 (1.5–1.8)	1.6 (1.4–1.7)^c^

Displayed values in this table are unweighted to present raw values (estimates for values in subsequent figures are weighted)

^a^ Corresponding 95% confidence intervals are shown for each variable

^b^ Adjusted for age, sex, and race/ethnicity

^c^ Mortality by diabetes status was projected using a Poisson model with calendar period as continuous variable, using publicly available linked mortality data from the National Center for Health Statistics [8, 9]

We used a multistate model with three states (i.e., without diabetes, with diabetes, death) to estimate LR and YPLL. The age-specific 1-year transition rates without recession among those three states were 1) age-specific incidence of diabetes, 2) all-cause mortality among individuals with diabetes, and 3) all-cause mortality among individuals without diabetes. We estimated mortality using adults 18 years and older. The incidence rates were estimated using participants aged 18 to 84 based on the average life expectancy in the United States and the expected plateau of LR [12, 13]. We assumed a stochastic process with a Markov property, which depends only on the current state, not on prior events. Starting from a given age (*a*, year), the standard stochastic theory and approximate integral of survival function (*S*), incidence (*I*) and mortality (*M*) among individuals with diabetes (*M*_*1*_) and mortality among individuals without diabetes (*M*_*0*_) by discrete time (*t*, year) were used to calculate the LR and YPLL. The LR and YPLL from a given age without diabetes up to age 84 were calculated as shown in **S2 Fig**. To represent a measure of overall public health burden, years spent with diabetes was estimated in the entire sample using the Sullivan formula [14], as a weighted average of individuals with and without diabetes and therefore was not contingent on developing diabetes. All estimates are calculated from the period perspective rather than from the cohort perspective. For example, YPLL are estimated as period expected years of life lost, as defined previously [15]. Additionally, all estimates account for different lengths of time in each survey cycle period. Variance estimates for each metric were estimated using Monte Carlo simulation with 1,000 replications and the lowest 2.5% and highest 97.5% of simulated estimates served as the lower and upper bounds of a 95% confidence interval (CI). Estimates for each metric are made up to age 60 because estimates for older ages may have high variability. Estimates with a *p* value less than 0.05 were considered to be statistically significant. For each of the three metrics (LR, YPLL, and years spent with diabetes), p-values from a Spearman’s Rho test were estimated to assess any monotonic trend across the five serial cross-sectional time periods. These p-values are indicated as “p-value for trend” in each of the figures and supplementary tables. Other comparisons of time periods and subgroups are described conservatively using non-overlap of confidence intervals. Prevalence, incidence, and mortality analyses were conducted using Stata (version 16.1, StataCorp, College Station, Texas). R (www.r-project.org) statistical software was used to estimate each of the three primary metrics.

## Results

Participants from the 1997 to 2018 NHIS cycles included 653,811 men and non-pregnant women aged 18 years and older with (n = 56,029) or without (n = 597,782) self-reported diagnosed diabetes. Individuals who reported a duration of diabetes of greater than one year were excluded from incidence analyses (n = 49,671). **Table 1** shows baseline characteristics, estimates of prevalent and incident diabetes, and mortality rates by time period. The mean age of adults was 44.1 years (95% CI: 43.9–44.3) in 1997–1999 and 46.5 years (95% CI: 46.3–46.7) in 2015–2018. The proportion of non-Hispanic Whites decreased from 74.7% (95% CI: 74.0–75.3%) in 1997–1999 to 64.3% (95% CI: 63.2–65.5) in 2015–2018. On average, adults had higher levels of education in later time periods. While crude prevalence of diabetes increased steadily over time, incidence peaked during the 2005–2009 time period. Crude all-cause mortality decreased over time among adults with and without diabetes. After adjustment for age, sex, and race/ethnicity, the rate ratio for mortality (with vs. without diabetes), decreased by 15.8% from 1.9 (95% CI: 1.8–2.0) in 1997–1999 to 1.6 (95% CI: 1.4–1.7) in 2015–2018.

**Fig 1:**
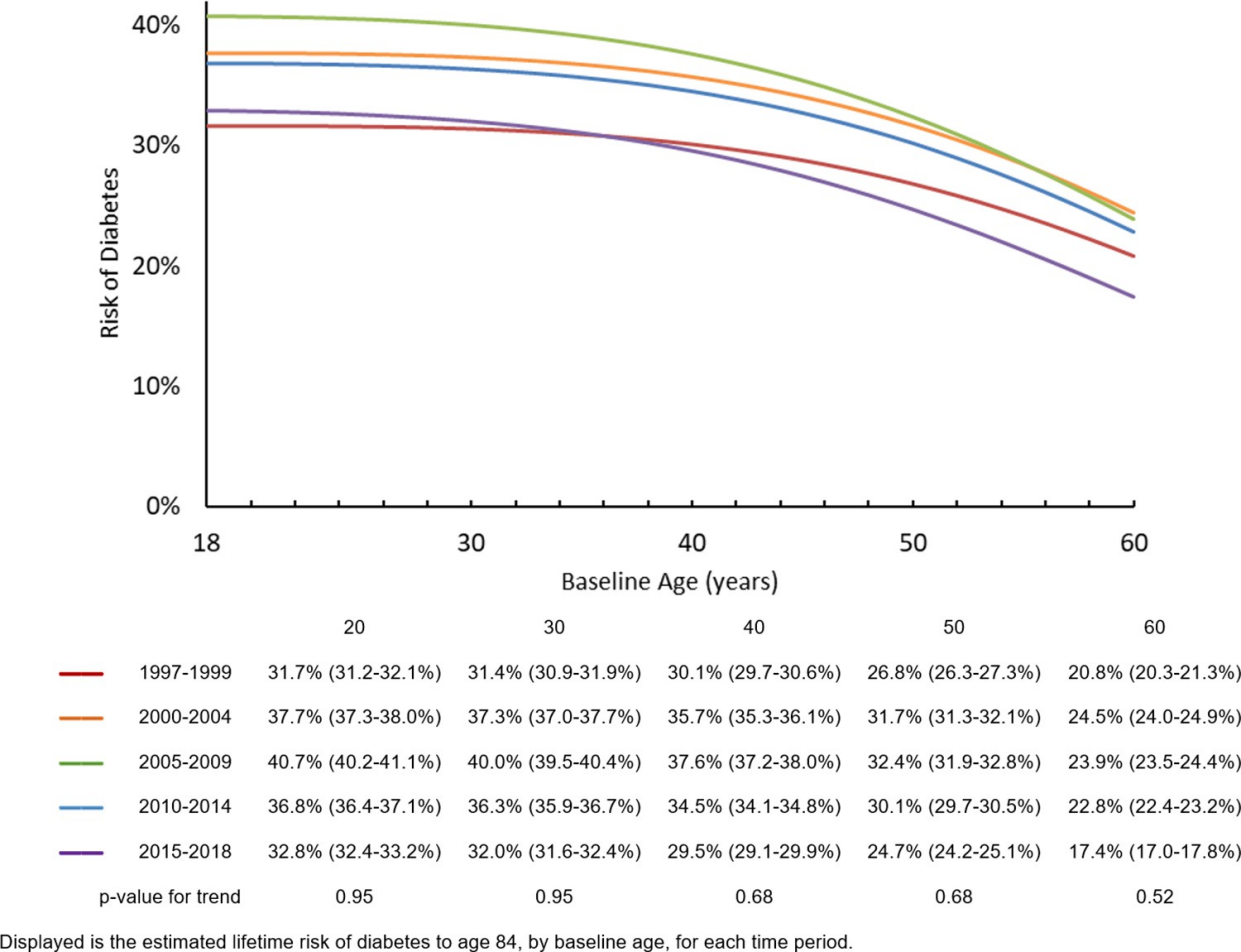
Lifetime risk of diabetes from age 18 to 84, by baseline age and time period.

**Fig 1** shows the LR to age 84 of diabetes for adults with a baseline age of 18–60 years old, by time period and different baseline ages (LR over age 60 is now shown due to high variability). Across baseline ages, LR increased from the 1997–1999 time period, peaked in the 2005–2009 or 2000–2004 time period, and decreased afterwards. For example, for adults 20 years of age, the estimated LR of diabetes in 1997–1999 was 31.7% (95% CI: 31.2–32.1%), meaning the average risk of developing diabetes for a 20 year old adult in this time period, until age 84 (approximate average life expectancy and plateau of LR for diabetes), was 31.7%. The LR then peaked in 2005–2009 at 40.7% (95% CI: 40.2–41.1%), and decreased afterwards, reaching 32.8% (95% CI: 32.4–33.2%) in 2015–2018. For adults 60 years of age, LR in 1997–1999 was 20.8% (95% CI: 20.3–21.3%), peaked in 2000–2004 (earlier than other age groups) to 24.5% (95% CI: 24.0–24.9%), and declined to 17.4% (95% CI: 17.0–17.8%) by 2015–2018, slightly below the LR during 1997–1999. LR for diabetes was similar across both sexes (**S1 Table**), but differed by race/ethnicity, with the highest average risk among Non-Hispanic Blacks and Hispanics, followed by adults of other races, and Non-Hispanic Whites having the lowest LR (**S2 Table**). No significant trend in LR was observed across the time periods overall, by sex, or by race/ethnicity.

**Fig 2:**
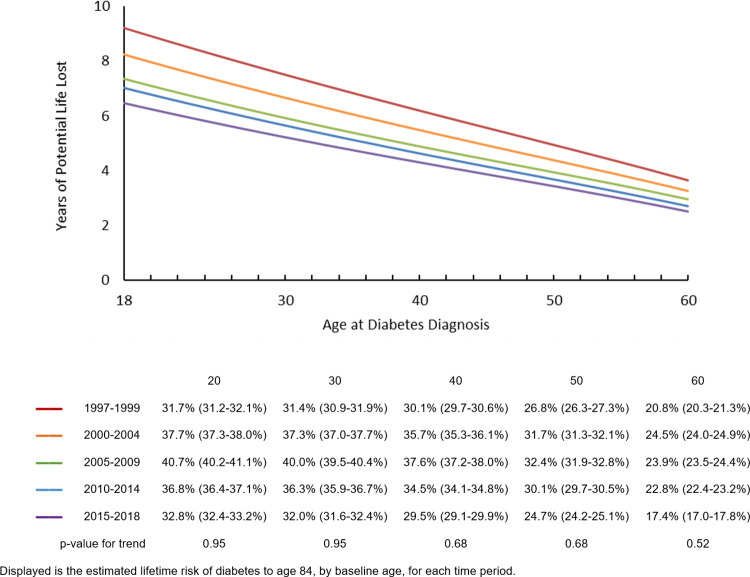
Years of potential life lost to age 84, by age at diabetes diagnosis and time period.

**Fig 2** shows the YPLL to age 84 by age at diabetes diagnosis from age 18 to 60, and by time period. At each age, YPLL decreased across time periods. For example, during the earliest time period in 1997–1999, a person diagnosed with diabetes at age 20 was estimated to lose on average 8.9 (95% CI: 8.7–9.1) years of potential life due to diabetes, decreasing to an average of 6.2 (95% CI: 6.1–6.4) years in 2015–2018. As expected, YPLL decreased as age increased, with a person diagnosed at 60 years during 2015–2018 losing an average 2.5 (95% CI: 2.4–2.6) years of potential life. Trends for YPLL followed a similar pattern for each sex, with a significant decline across time periods (**S3 Table**) and a slightly higher YPLL for men at younger ages compared to women. Trends by race/ethnicity over time did not show any consistent pattern and were not statistically significant (**S4 Table**).

**Fig 3:**
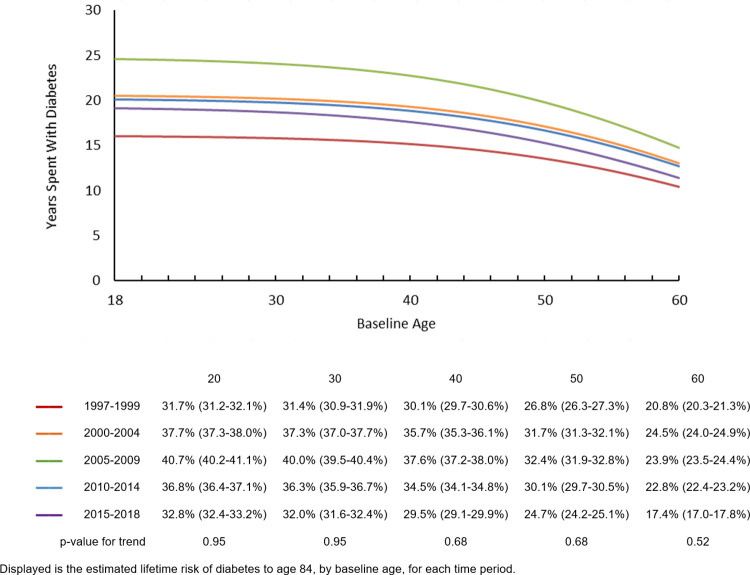
Years spent with diabetes, by baseline age and time period.

**Fig 3** shows the years spent with diabetes from various ages until age 84 by time period. This metric was estimated in the entire sample, not contingent on diabetes status and therefore was equivalent to a weighted average of the number of years spent with diabetes, among individuals who did and did not develop diabetes. Years spent with diabetes increased from 1997–1999 to 2005–2009, then decreased through 2015–2018. For example, during 1997–1999, the average number of years spent with diabetes between age 20 and 84 years by a person in the general population, not contingent on diabetes status, was 16.0 (95% CI: 15.7–16.3) years. This number increased to 24.6 (95% CI: 24.1–25.0) by 2005–2009, and then decreased to 19.1 (95% CI: 18.6–19.5) by 2015–2018. As age increased, remaining years spent with diabetes decreased, with an accelerated decrease in later stages of life. When stratified by sex or race/ethnicity, a similar trend was observed across age and time periods. Men on average had a greater number of years spent with diabetes compared to women (**S5 Table**). Across all age and time periods, non-Hispanic White adults had the lowest mean number of years spent with diabetes, while non-Hispanic Black adults had the highest (**S6 Table**).

## Discussion

Over a 22-year period, in a nationally representative sample of adults in the United States, the overall adult LR of diabetes from age 20 to 84 years peaked at 40.7% in 2005–2009 and then declined to 32.8% in 2015–2018. These data suggest that in recent years, about one-third of the population is being diagnosed with diabetes during their adult lifetime.

The decrease in LR of diabetes since 2010 parallels the decrease in diabetes incidence among US adults [16]. While decreasing mortality may increase LR by extending the amount of time an individual is at risk, incidence predominately drives LR, as reflected in these findings. Moreover, it is possible that the preferential use of HbA1c as a diagnostic test since 2010 may have contributed to a lower LR by underestimating diabetes diagnoses [17]. A multi-country analysis of trends in diabetes incidence, however, found no temporal relationship between the formal introduction of HbA1c and the decline in diabetes incidence across 21 countries [16]. Although we cannot empirically evaluate specific potential causes, improvements in physical activity [18], decreases in sedentary activity [19], and modest improvements in diet quality [20] may have ameliorated the risk of diabetes at the population level. On the other hand, the prevalence of obesity and severe obesity have continued to increase since 1999 in the United States, particularly among non-White populations [21] and the age-adjusted prevalence of prediabetes has remained relatively constant since 2005 [1], Thus, while the secular decline in LR of diabetes may reflect a positive balance of favorable changes at the population level, it represents nonetheless a crude indicator of the complex interplay between preventive measures, demographic changes, and detection bias.

Adult LR remained high for non-Hispanic Black and Hispanic adults across all time periods, particularly for individuals 30 years and younger, in whom LR remained near or above 40% throughout the study period. These patterns are in line with results from a population-based registry study, in which the adjusted incidence of type 1 and type 2 diabetes increased more steeply from 2002 to 2015 among non-White than among White youth [22], Although specific reasons for increasing rates in type 1 diabetes are not known, the continuous increase in body mass index is a powerful driver for type 2 diabetes incidence, with a stronger association reported between overweight and type 2 diabetes diagnosis at ages 30 to 60 years than after 60 years of age [23], Additionally, as the observed results are population-level averages, it is possible there are other unobserved risk profiles based on race and ethnicity. For example, a more detailed classification may show large differences in risk between subpopulations within Hispanic and Asian populations as well as high risk in Native Americans [24].

YPLL decreased linearly over time regardless of sex and race/ethnicity, paralleling decreasing mortality rates during the study period and improvements in some aspects of diabetes management such as lipid and blood pressure control [25]. A sex disparity was observed with men having a higher YPLL compared to women at earlier ages of diabetes onset. This may be attributed at least in part to an increased risk of cardiovascular disease in men compared to pre-menopausal women, and the more similar risks in older age groups between men and post-menopausal women [26].

The years spent with diabetes in the general population begin to decline in the most recent time periods, though the estimates remain high. For each five-year increase in duration of diabetes, a previous study reported approximately a 25% and 50% increased risk of microvascular and macrovascular events, respectively [27]. Given the health and financial burdens of diabetes complications, these figures further emphasize the importance of continuing efforts in both prevention and delay of type 2 diabetes.

While the observed decreases in LR and YPLL are promising, the impact of COVID-19 may disrupt current trends. For example, LR may be expected to decrease among older adults because of increased mortality due to COVID-19 primarily affecting older adults and consequently acting as a competing risk. YPLL may also increase through both direct and indirect impacts of COVID-19. Consistent evidence suggests individuals with diabetes are at increased risk of severe COVID-19 outcomes and mortality [28]. Additionally, measures to mitigate SARS-CoV2 transmission can present challenges to optimal diabetes management, resulting in less frequent HbA1c testing [29] and decreases in physical activity and diet quality [30], which may result in a higher risk of complications and subsequent mortality risk. Conversely, any negative impact of COVID-19 may be attenuated by effective emerging therapeutic agents such as inhibitors of sodium-glucose co-transporters 1 and 2 (SGLT1, SGLT2).

LR can be estimated using either longitudinal cohorts or serial cross-sectional data. Using longitudinal data is subject to particular challenges such as the difficulty of following individuals over their lifetime, the self-selection of participants into the cohort, and the applicability of decades-old data from early life to the current population. In contrast, strengths of using serial cross-sectional data are the more feasible approach and use of more current data. Moreover, results from a nationally representative sample such as NHIS are more applicable for estimating risk in the general population. The estimates of LR, YPLL, and years spent with diabetes are also subject to certain limitations. The current approach assumes current age-specific incidence and mortality remain consistent in the future. Similarly, because data were based on adults from 18 years of age, we assume no changes in risk or mortality occur before this age. However, as the majority of both risk and mortality occur at later ages, the potential impact on estimates is likely minimal. Likewise, estimates of LR, YPLL, and years spent with diabetes in the current study may be underestimated compared to previous publications that have extrapolated beyond age 84. In addition, mortality rates were modelled for the first and last three years of the study period. Combining estimates from models may have resulted in increased statistical uncertainty in detecting trends across time periods. Finally, incidence estimates were based on self-report and do not include undiagnosed diabetes, which if included, may increase LR.

## Conclusion

In this nationally representative study of adults in the United States from 1997 to 2018, we observed a decrease in LR, YPLL, and years spent with diabetes in the most recent time periods. While these results are promising, continuing efforts can help prevent incident diabetes, improve diabetes management, as well as reduce disparities.

## Supporting information

S1 Table Lifetime risk of diabetes by baseline age, time period and sex. (DOCX)

S2 Table Lifetime risk of diabetes by baseline age, time period and race/ethnicity. (DOCX)

S3 Table Years of potential life lost, by age at diabetes diagnosis, time period, and sex. (DOCX)

S4 Table Years of potential life lost, by age at diabetes diagnosis, time period, and race/ethnicity. (DOCX)

S5 Table Years of life spent with diabetes, by baseline age, time period, and sex. (DOCX)

S6 Table Years of life spent with diabetes, by baseline age, time period, and race/ethnicity. (DOCX)

S1 Fig Flow chart for study sample. (TIF)

S2 Fig Formula used to derive lifetime risk. (TIF)
