# Correction: Trends in lifetime risk and years of potential life lost from diabetes in the United States, 1997–2018

**DOI:** 10.1371/journal.pone.0326955

**Published:** 2025-06-23

**Authors:** Alain K. Koyama, Yiling J. Cheng, Ralph Brinks, Hui Xie, Edward W. Gregg, Annika Hoyer, Meda E. Pavkov, Giuseppina Imperatore

Figs 2 and 3 are incorrect. The numbers indicated in the images are duplicated from Fig 1. Please see the correct Figs 2 and 3 here.

**Fig 2:**
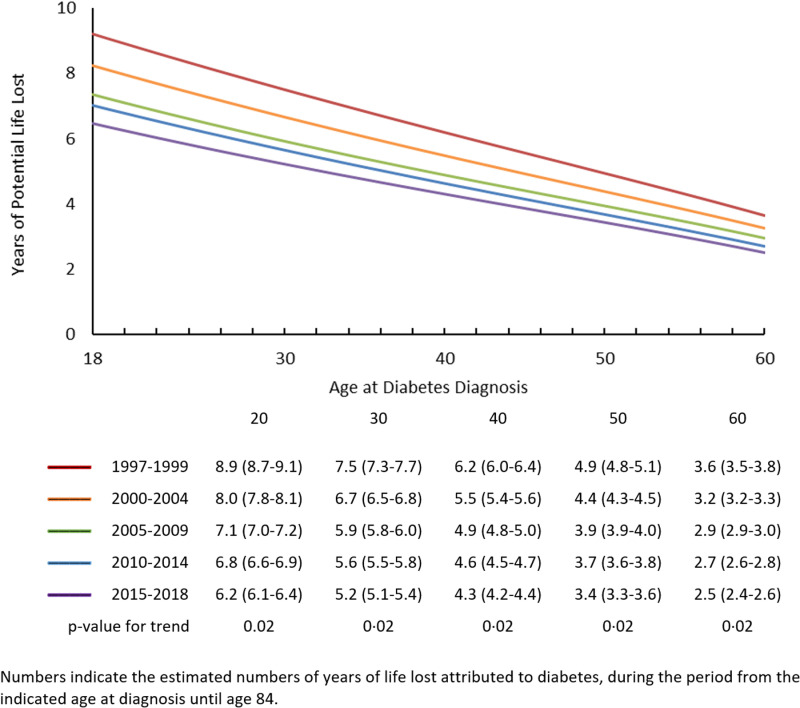
Years of potential life lost to age 84, by age at diabetes diagnosis and time period.

**Fig 3:**
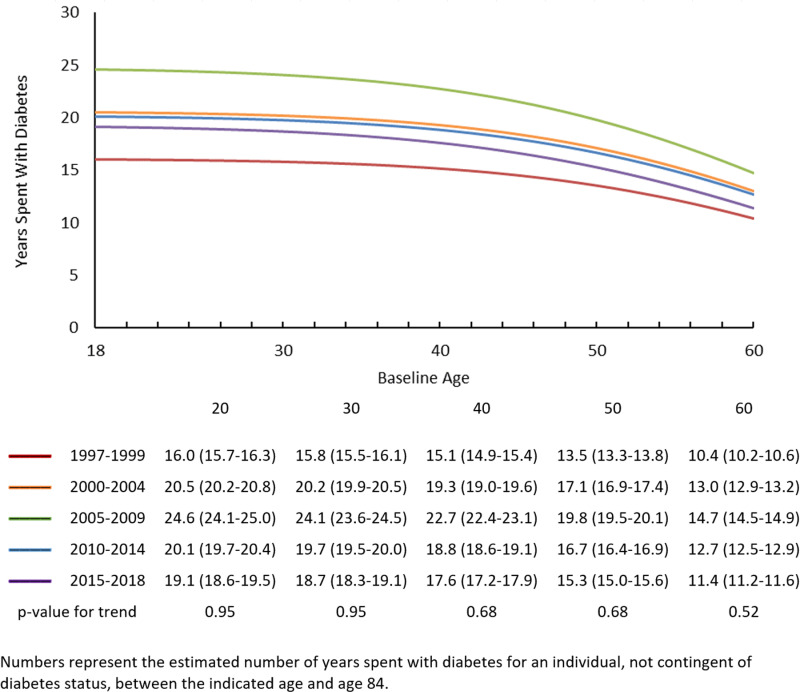
Years spent with diabetes, by baseline age and time period.
