# Supplementary material for: Trends in lifetime risk and years of potential life lost from diabetes in the United States, 1997–2018
Source: PLoS One. 2022 May 24;17(5):e0268805. doi: 10.1371/journal.pone.0268805 (PMC9129010; doi:10.1371/journal.pone.0268805)
Supplement: S1 Table — (DOCX) [file pone.0268805.s001.docx]

**S1 Table – Lifetime Risk of Diabetes by Baseline Age, Time Period and Sex**

|  | **Men** | | | | |  | **Women** | | | | |
| --- | --- | --- | --- | --- | --- | --- | --- | --- | --- | --- | --- |
|  | **20** | **30** | **40** | **50** | **60** |  | **20** | **30** | **40** | **50** | **60** |
| **1997-1999** | 30·4% (29·8-30·9%) | 30·3% (29·7-30·8%) | 29·2% (28·6-29·7%) | 26·2% (25·5-26·8%) | 20·5% (19·8-21·1%) |  | 32·8% (32·3-33·4%) | 32·5% (31·9-33·0%) | 31·0% (30·4-31·5%) | 27·4% (26·8-27·9%) | 21·0% (20·5-21·6%) |
| **2000-2004** | 38·0% (37·5-38·4%) | 37·8% (37·3-38·2%) | 36·3% (35·8-36·8%) | 32·5% (32·0-33·0%) | 25·4% (24·8-25·9%) |  | 37·3% (36·8-37·7%) | 36·8% (36·3-37·2%) | 35·0% (34·5-35·4%) | 30·8% (30·2-31·2%) | 23·4% (22·9-23·9%) |
| **2005-2009** | 40·9% (40·4-41·4%) | 40·4% (39·8-40·9%) | 38·2% (37·7-38·7%) | 33·2% (32·6-33·8%) | 24·8% (24·3-25·4%) |  | 40·3% (39·7-40·8%) | 39·4% (38·9-40·0%) | 36·8% (36·3-37·3%) | 31·4% (30·9-31·9%) | 22·9% (22·3-23·5%) |
| **2010-2014** | 35·3% (34·8-35·7%) | 34·9% (34·5-35·4%) | 33·3% (32·9-33·8%) | 29·4% (28·9-29·8%) | 22·4% (21·9-22·9%) |  | 38·2% (37·7-38·7%) | 37·6% (37·1-38·0%) | 35·5% (35·0-35·9%) | 30·8% (30·3-31·3%) | 23·1% (22·6-23·6%) |
| **2015-2018** | 32·6% (32·0-33·0%) | 31·9% (31·3-32·4%) | 29·7% (29·1-30·1%) | 25·0% (24·5-25·5%) | 17·9% (17·4-18·3%) |  | 32·9% (32·4-33·5%) | 31·9% (31·4-32·5%) | 29·3% (28·8-29·8%) | 24·3% (23·7-24·8%) | 16·9% (16·4-17·4%) |
| **p-value for trend** | 0·95 | 0·95 | 0·68 | 0·68 | 0·52 |  | 0·68 | 0·95 | 0·95 | 0·68 | 0·68 |
