# Supplementary material for: Trends in lifetime risk and years of potential life lost from diabetes in the United States, 1997–2018
Source: PLoS One. 2022 May 24;17(5):e0268805. doi: 10.1371/journal.pone.0268805 (PMC9129010; doi:10.1371/journal.pone.0268805)
Supplement: S2 Table — (DOCX) [file pone.0268805.s002.docx]

**S2 Table – Lifetime Risk of Diabetes by Baseline Age, Time Period and Race/Ethnicity**

|  | **Non-Hispanic Whites** | | | | |  | **Non-Hispanic Blacks** | | | | | |
| --- | --- | --- | --- | --- | --- | --- | --- | --- | --- | --- | --- | --- |
|  | **20** | **30** | **40** | **50** | **60** |  | **20** | **30** | **40** | **50** | **60** |  |
| **1997-1999** | 26·4% (25·9-26·9%) | 26·1% (25·6-26·6%) | 25·0% (24·5-25·5%) | 22·2% (21·7-22·7%) | 17·1% (16·6-17·6%) |  | 43·2% (42·4-44·1%) | 43·0% (42·1-43·8%) | 41·3% (40·4-42·2%) | 36·9% (35·9-37·9%) | 28·8% (27·7-29·8%) |  |
| **2000-2004** | 32·9% (32·5-33·2%) | 32·5% (32·1-32·8%) | 31·0% (30·6-31·4%) | 27·4% (27·0-27·8%) | 21·1% (20·7-21·4%) |  | 45·6% (44·9-46·5%) | 45·3% (44·5-46·1%) | 43·4% (42·6-44·2%) | 38·5% (37·6-39·4%) | 29·8% (28·8-30·7%) |  |
| **2005-2009** | 37·7% (37·2-38·2%) | 37·0% (36·5-37·5%) | 34·7% (34·2-35·2%) | 29·9% (29·4-30·4%) | 22·0% (21·5-22·5%) |  | 46·5% (45·5-47·5%) | 45·7% (44·7-46·7%) | 42·9% (41·9-44·0%) | 36·9% (35·8-38·1%) | 27·1% (26·0-28·2%) |  |
| **2010-2014** | 32·6% (32·2-33·0%) | 32·1% (31·7-32·5%) | 30·4% (30·0-30·8%) | 26·5% (26·1-27·0%) | 20·0% (19·5-20·4%) |  | 45·5% (44·9-46·1%) | 45·0% (44·4-45·6%) | 42·8% (42·1-43·4%) | 37·5% (36·7-38·2%) | 28·3% (27·6-29·1%) |  |
| **2015-2018** | 28·6% (28·2-29·0%) | 27·8% (27·4-28·2%) | 25·7% (25·2-26·1%) | 21·4% (21·0-21·8%) | 15·1% (14·7-15·5%) |  | 39·7% (38·8-40·6%) | 38·7% (37·8-39·6%) | 35·7% (34·7-36·7%) | 29·8% (28·8-30·7%) | 20·9% (19·9-21·8%) |  |
| **p-value for trend** | 0·95 | 0·95 | 0·95 | 0·68 | 0·68 |  | 0·68 | 0·68 | 0·52 | 0·45 | 0·13 |  |
|  | **Hispanic** | | | | |  | **Other** | | | | | |
|  | **20** | **30** | **40** | **50** | **60** |  | **20** | **30** | **40** | **50** | **60** |  |
| **1997-1999** | 41·2% (40·2-42·1%) | 41·4% (40·4-42·3%) | 40·3% (39·2-41·3%) | 36·6% (35·4-37·7%) | 29·2% (27·9-30·4%) |  | 36·0% (33·8-37·9%) | 35·8% (33·6-37·7%) | 34·3% (32·1-36·3%) | 30·5% (28·2-32·6%) | 23·5% (21·1-25·6%) |  |
| **2000-2004** | 46·5% (45·7-47·4%) | 46·7% (45·8-47·6%) | 45·3% (44·4-46·2%) | 41·0% (40·0-42·1%) | 32·6% (31·5-33·7%) |  | 47·6% (46·2-49·0%) | 47·3% (45·9-48·7%) | 45·3% (43·9-46·9%) | 40·4% (38·6-42·0%) | 31·4% (29·6-33·2%) |  |
| **2005-2009** | 47·1% (46·3-47·9%) | 46·9% (46·0-47·6%) | 44·6% (43·7-45·5%) | 39·1% (38·1-40·0%) | 29·6% (28·6-30·6%) |  | 43·6% (41·9-45·2%) | 42·9% (41·2-44·5%) | 40·2% (38·5-41·8%) | 34·4% (32·7-36·1%) | 25·2% (23·7-26·7%) |  |
| **2010-2014** | 47·5% (46·7-48·3%) | 47·5% (46·7-48·3%) | 45·8% (44·9-46·6%) | 40·9% (40·0-41·9%) | 32·0% (31·0-33·0%) |  | 33·3% (32·2-34·4%) | 32·9% (31·7-34·0%) | 31·0% (29·9-32·1%) | 26·8% (25·6-28·0%) | 19·9% (18·6-21·0%) |  |
| **2015-2018** | 43·4% (42·3-44·4%) | 42·8% (41·7-43·9%) | 40·2% (38·9-41·3%) | 34·3% (33·1-35·5%) | 25·0% (23·9-26·3%) |  | 35·8% (34·6-37·1%) | 34·9% (33·7-36·1%) | 32·1% (30·8-33·3%) | 26·6% (25·3-27·9%) | 18·6% (17·3-19·9%) |  |
| **p-value for trend** | 0·52 | 0·52 | 0·95 | 0·68 | 0·68 |  | 0·35 | 0·35 | 0·35 | 0·23 | 0·23 |  |
