# Supplementary material for: Trends in lifetime risk and years of potential life lost from diabetes in the United States, 1997–2018
Source: PLoS One. 2022 May 24;17(5):e0268805. doi: 10.1371/journal.pone.0268805 (PMC9129010; doi:10.1371/journal.pone.0268805)
Supplement: S3 Table — (DOCX) [file pone.0268805.s003.docx]

**S3 Table – Years of Potential Life Lost, by Age at Diabetes Diagnosis, Time Period, and Sex**

|  | **Men** | | | | |  | **Women** | | | | |
| --- | --- | --- | --- | --- | --- | --- | --- | --- | --- | --- | --- |
|  | 20 | 30 | **40** | **50** | **60** |  | **20** | **30** | **40** | **50** | **60** |
| **1997-1999** | 9·3 (9·1-9·6) | 7·8 (7·6-8·0) | 6·3 (6·1-6·6) | 5·0 (4·8-5·2) | 3·6 (3·5-3·8) |  | 8·4 (8·2-8·6) | 7·2 (7·0-7·4) | 6·0 (5·8-6·2) | 4·9 (4·7-5·0) | 3·6 (3·5-3·8) |
| **2000-2004** | 8·3 (8·1-8·5) | 6·8 (6·7-7·0) | 5·5 (5·4-5·7) | 4·4 (4·3-4·5) | 3·2 (3·1-3·3) |  | 7·6 (7·4-7·8) | 6·4 (6·3-6·6) | 5·4 (5·2-5·5) | 4·4 (4·2-4·5) | 3·3 (3·2-3·4) |
| **2005-2009** | 7·4 (7·2-7·6) | 6·1 (6·0-6·2) | 4·9 (4·8-5·1) | 3·9 (3·8-4·0) | 2·9 (2·8-3·0) |  | 6·8 (6·6-6·9) | 5·7 (5·6-5·9) | 4·8 (4·7-4·9) | 3·9 (3·9-4·0) | 3·0 (2·9-3·1) |
| **2010-2014** | 7·1 (6·9-7·3) | 5·8 (5·7-6·0) | 4·7 (4·6-4·8) | 3·7 (3·6-3·8) | 2·7 (2·6-2·8) |  | 6·4 (6·2-6·5) | 5·4 (5·3-5·5) | 4·5 (4·4-4·6) | 3·6 (3·5-3·7) | 2·7 (2·6-2·8) |
| **2015-2018** | 6·5 (6·3-6·7) | 5·3 (5·2-5·5) | 4·3 (4·2-4·5) | 3·4 (3·2-3·5) | 2·4 (2·3-2·6) |  | 6·0 (5·8-6·1) | 5·1 (4·9-5·2) | 4·2 (4·1-4·4) | 3·4 (3·3-3·6) | 2·5 (2·4-2·7) |
| **p-value for trend** | 0·02 | 0·02 | 0·02 | 0·02 | 0·02 |  | 0·02 | 0·02 | 0·02 | 0·02 | 0·02 |
