# Supplementary material for: Trends in lifetime risk and years of potential life lost from diabetes in the United States, 1997–2018
Source: PLoS One. 2022 May 24;17(5):e0268805. doi: 10.1371/journal.pone.0268805 (PMC9129010; doi:10.1371/journal.pone.0268805)
Supplement: S4 Table — (DOCX) [file pone.0268805.s004.docx]

**S4 Table – Years of Potential Life Lost, by Age at Diabetes Diagnosis, Time Period, and Race/Ethnicity**

|  | **Non-Hispanic Whites** | | | | |  | **Non-Hispanic Blacks** | | | | | |
| --- | --- | --- | --- | --- | --- | --- | --- | --- | --- | --- | --- | --- |
|  | **20** | **30** | **40** | **50** | **60** |  | **20** | **30** | **40** | **50** | **60** |  |
| **1997-1999** | 9·4 (9·1-9·6) | 8·1 (7·9-8·3) | 6·9 (6·7-7·1) | 5·6 (5·4-5·7) | 4·2 (4·0-4·3) |  | 8·6 (8·2-8·9) | 7·1 (6·8-7·4) | 5·7 (5·4-6·0) | 4·5 (4·2-4·7) | 3·3 (3·0-3·5) |  |
| **2000-2004** | 8·4 (8·2-8·6) | 7·2 (7·1-7·4) | 6·1 (6·0-6·2) | 5·0 (4·9-5·1) | 3·7 (3·6-3·8) |  | 7·8 (7·5-8·1) | 6·4 (6·1-6·6) | 5·1 (4·9-5·3) | 4·0 (3·8-4·2) | 2·9 (2·8-3·1) |  |
| **2005-2009** | 10·6 (10·3-10·8) | 9·0 (8·8-9·2) | 7·5 (7·3-7·7) | 6·1 (5·9-6·2) | 4·6 (4·4-4·7) |  | 4·1 (3·9-4·3) | 3·4 (3·2-3·5) | 2·7 (2·6-2·8) | 2·2 (2·1-2·2) | 1·6 (1·5-1·7) |  |
| **2010-2014** | 7·2 (7·0-7·3) | 6·2 (6·1-6·3) | 5·2 (5·1-5·3) | 4·2 (4·1-4·3) | 3·1 (3·1-3·2) |  | 6·6 (6·3-6·8) | 5·3 (5·1-5·5) | 4·3 (4·1-4·4) | 3·3 (3·2-3·5) | 2·4 (2·2-2·5) |  |
| **2015-2018** | 6·7 (6·5-6·9) | 5·8 (5·6-5·9) | 4·9 (4·7-5·0) | 4·0 (3·8-4·1) | 2·9 (2·8-3·1) |  | 6·1 (5·9-6·3) | 5·0 (4·8-5·2) | 4·0 (3·8-4·2) | 3·1 (2·9-3·3) | 2·2 (2·0-2·4) |  |
| **p-value for trend** | 0·23 | 0·23 | 0·23 | 0·23 | 0·18 |  | 0·23 | 0·23 | 0·23 | 0·23 | 0·23 |  |
|  | **Hispanic** | | | | |  | **Other** | | | | | |
|  | **20** | **30** | **40** | **50** | **60** |  | **20** | **30** | **40** | **50** | **60** |  |
| **1997-1999** | 8·5 (8·2-8·8) | 6·3 (6·1-6·6) | 4·6 (4·4-4·8) | 3·2 (3·0-3·4) | 2·1 (1·9-2·2) |  | 4·9 (4·6-5·3) | 3·7 (3·4-4·1) | 2·7 (2·4-3·0) | 1·9 (1·6-2·1) | 1·1 (0·9-1·4) |  |
| **2000-2004** | 3·5 (3·2-3·8) | 2·7 (2·5-3·0) | 2·1 (1·9-2·4) | 1·6 (1·4-1·8) | 1·2 (1·0-1·3) |  | 8·2 (7·9-8·5) | 5·8 (5·6-6·0) | 4·0 (3·8-4·2) | 2·6 (2·4-2·7) | 1·5 (1·3-1·6) |  |
| **2005-2009** | 6·8 (6·5-7·0) | 4·9 (4·7-5·1) | 3·5 (3·4-3·6) | 2·4 (2·3-2·6) | 1·6 (1·4-1·7) |  | 3·6 (3·3-3·9) | 2·6 (2·4-2·9) | 1·9 (1·6-2·1) | 1·2 (1·0-1·4) | 0·7 (0·5-0·9) |  |
| **2010-2014** | 6·2 (6·0-6·5) | 4·5 (4·3-4·6) | 3·1 (3·0-3·3) | 2·1 (2·0-2·2) | 1·3 (1·2-1·4) |  | 3·3 (3·0-3·6) | 2·4 (2·1-2·7) | 1·6 (1·4-1·9) | 1·0 (0·8-1·2) | 0·5 (0·3-0·7) |  |
| **2015-2018** | 5·6 (5·3-5·9) | 4·0 (3·8-4·2) | 2·8 (2·6-3·0) | 1·9 (1·7-2·1) | 1·1 (1·0-1·3) |  | 2·8 (2·4-3·1) | 1·9 (1·6-2·2) | 1·3 (1·0-1·5) | 0·7 (0·5-1·0) | 0·3 (0·1-0·5) |  |
| **p-value for trend** | 0·52 | 0·52 | 0·52 | 0·52 | 0·23 |  | 0·08 | 0·08 | 0·08 | 0·08 | 0·08 |  |
