# Supplementary material for: Trends in lifetime risk and years of potential life lost from diabetes in the United States, 1997–2018
Source: PLoS One. 2022 May 24;17(5):e0268805. doi: 10.1371/journal.pone.0268805 (PMC9129010; doi:10.1371/journal.pone.0268805)
Supplement: S5 Table — (DOCX) [file pone.0268805.s005.docx]

**S5 Table – Years of Life Spent With Diabetes, by Baseline Age, Time Period, and Sex**

|  | **Men** | | | | |  | **Women** | | | | |
| --- | --- | --- | --- | --- | --- | --- | --- | --- | --- | --- | --- |
|  | **20** | **30** | **40** | **50** | **60** |  | **20** | **30** | **40** | **50** | **60** |
| **1997-1999** | 17·6 (17·1-18·0) | 17·4 (16·9-17·8) | 16·6 (16·2-17·1) | 14·8 (14·4-15·2) | 11·3 (11·0-11·6) |  | 14·6 (14·2-14·9) | 14·4 (14·1-14·7) | 13·8 (13·5-14·1) | 12·4 (12·1-12·6) | 9·6 (9·4-9·8) |
| **2000-2004** | 24·1 (23·6-24·6) | 23·7 (23·3-24·2) | 22·7 (22·2-23·1) | 20·0 (19·7-20·5) | 15·1 (14·8-15·4) |  | 17·6 (17·3-17·9) | 17·3 (17·0-17·7) | 16·6 (16·3-16·9) | 14·7 (14·5-15·0) | 11·3 (11·1-11·5) |
| **2005-2009** | 28·6 (28·0-29·3) | 28·1 (27·5-28·7) | 26·5 (26·0-27·1) | 23·0 (22·5-23·4) | 16·9 (16·6-17·2) |  | 21·2 (20·7-21·7) | 20·8 (20·3-21·3) | 19·7 (19·2-20·1) | 17·1 (16·8-17·5) | 12·9 (12·6-13·1) |
| **2010-2014** | 21·8 (21·4-22·3) | 21·5 (21·1-21·9) | 20·5 (20·1-20·9) | 18·1 (17·8-18·4) | 13·7 (13·5-14·0) |  | 18·5 (18·1-18·8) | 18·2 (17·9-18·5) | 17·3 (17·0-17·6) | 15·4 (15·1-15·6) | 11·8 (11·6-12·0) |
| **2015-2018** | 21·6 (21·0-22·2) | 21·1 (20·6-21·7) | 19·9 (19·4-20·4) | 17·2 (16·8-17·6) | 12·8 (12·5-13·0) |  | 17·0 (16·5-17·4) | 16·6 (16·2-17·0) | 15·6 (15·3-16·0) | 13·6 (13·3-13·9) | 10·2 (10·0-10·5) |
| **p-value for trend** | 0·95 | 0·95 | 0·95 | 0·95 | 0·95 |  | 0·68 | 0·68 | 0·68 | 0·68 | 0·68 |
