# Supplementary material for: Trends in lifetime risk and years of potential life lost from diabetes in the United States, 1997–2018
Source: PLoS One. 2022 May 24;17(5):e0268805. doi: 10.1371/journal.pone.0268805 (PMC9129010; doi:10.1371/journal.pone.0268805)
Supplement: S6 Table — (DOCX) [file pone.0268805.s006.docx]

**S6 Table – Years of Life Spent With Diabetes, by Baseline Age, Time Period, and Race/Ethnicity**

|  | **Non-Hispanic Whites** | | | | |  | **Non-Hispanic Blacks** | | | | | |
| --- | --- | --- | --- | --- | --- | --- | --- | --- | --- | --- | --- | --- |
|  | **20** | **30** | **40** | **50** | **60** |  | **20** | **30** | **40** | **50** | **60** |  |
| **1997-1999** | 12·0 (11·7-12·3) | 11·9 (11·6-12·1) | 11·4 (11·2-11·7) | 10·2 (10·0-10·5) | 7·9 (7·7-8·1) |  | 36·8 (35·4-38·3) | 36·1 (34·8-37·6) | 34·3 (33·0-35·6) | 29·7 (28·6-30·9) | 21·6 (20·8-22·4) |  |
| **2000-2004** | 16·3 (16·0-16·5) | 16·1 (15·8-16·3) | 15·4 (15·2-15·6) | 13·7 (13·5-13·9) | 10·5 (10·4-10·7) |  | 39·8 (38·4-41·2) | 39·1 (37·7-40·5) | 36·9 (35·6-38·2) | 31·9 (30·8-33·0) | 23·1 (22·3-23·8) |  |
| **2005-2009** | 21·0 (20·5-21·5) | 20·6 (20·1-21·1) | 19·5 (19·0-19·9) | 16·8 (16·5-17·2) | 12·3 (12·1-12·6) |  | 41·5 (40·2-42·8) | 40·6 (39·4-41·8) | 38·0 (36·9-39·2) | 32·5 (31·5-33·4) | 23·5 (22·9-24·2) |  |
| **2010-2014** | 16·4 (16·1-16·8) | 16·2 (15·9-16·5) | 15·5 (15·2-15·8) | 13·8 (13·5-14·0) | 10·5 (10·4-10·7) |  | 38·7 (37·6-39·8) | 37·9 (36·9-39·0) | 35·7 (34·8-36·7) | 30·8 (30·0-31·6) | 22·3 (21·8-22·9) |  |
| **2015-2018** | 15·4 (15·1-15·9) | 15·1 (14·8-15·5) | 14·3 (14·0-14·7) | 12·5 (12·2-12·8) | 9·4 (9·2-9·6) |  | 34·5 (33·1-35·9) | 33·6 (32·3-34·9) | 31·3 (30·1-32·4) | 26·5 (25·5-27·4) | 18·9 (18·2-19·6) |  |
| **p-value for trend** | 0·68 | 0·68 | 0·68 | 0·68 | 0·68 |  | 0·68 | 0·68 | 0·68 | 0·68 | 0·68 |  |
|  | **Hispanic** | | | | |  | **Other** | | | | | |
|  | **20** | **30** | **40** | **50** | **60** |  | **20** | **30** | **40** | **50** | **60** |  |
| **1997-1999** | 19·7 (19·0-20·3) | 19·4 (18·8-20·0) | 18·6 (18·0-19·2) | 16·7 (16·2-17·2) | 13·1 (12·6-13·5) |  | 18·1 (16·8-19·3) | 17·9 (16·6-19·1) | 17·1 (15·9-18·3) | 15·4 (14·3-16·4) | 12·1 (11·2-12·8) |  |
| **2000-2004** | 23·7 (23·0-24·3) | 23·3 (22·7-24·0) | 22·3 (21·7-22·9) | 19·9 (19·4-20·5) | 15·5 (15·1-16·0) |  | 25·3 (24·3-26·3) | 24·9 (23·9-25·9) | 23·7 (22·8-24·6) | 20·9 (20·2-21·7) | 16·1 (15·5-16·7) |  |
| **2005-2009** | 25·5 (24·8-26·2) | 25·0 (24·3-25·6) | 23·6 (23·0-24·2) | 20·6 (20·1-21·1) | 15·7 (15·3-16·0) |  | 24·7 (23·1-26·2) | 24·2 (22·7-25·6) | 22·9 (21·5-24·1) | 20·0 (18·9-21·0) | 15·2 (14·5-15·9) |  |
| **2010-2014** | 24·2 (23·6-24·8) | 23·8 (23·2-24·3) | 22·6 (22·1-23·2) | 20·0 (19·6-20·5) | 15·5 (15·1-15·8) |  | 17·1 (16·4-17·8) | 16·8 (16·1-17·5) | 16·1 (15·4-16·7) | 14·3 (13·7-14·8) | 11·2 (10·7-11·6) |  |
| **2015-2018** | 23·5 (22·7-24·3) | 23·0 (22·2-23·8) | 21·6 (20·9-22·3) | 18·8 (18·2-19·4) | 14·3 (13·8-14·7) |  | 20·2 (19·3-21·2) | 19·8 (18·9-20·7) | 18·6 (17·9-19·4) | 16·3 (15·6-16·9) | 12·4 (11·9-12·9) |  |
| **p-value for trend** | 0·68 | 0·68 | 0·68 | 0·68 | 0·95 |  | 0·78 | 0·78 | 0·78 | 0·78 | 0·78 |  |
