# Supplementary figures and images for: Trends in lifetime risk and years of potential life lost from diabetes in the United States, 1997–2018
Source: PLoS One. 2022 May 24;17(5):e0268805. doi: 10.1371/journal.pone.0268805 (PMC9129010; doi:10.1371/journal.pone.0268805)

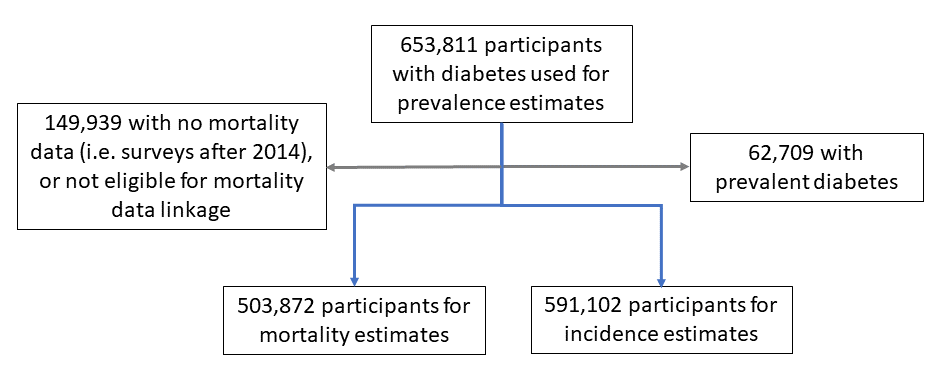

Supplement: S1 Fig — (TIF) [file pone.0268805.s007.tif]

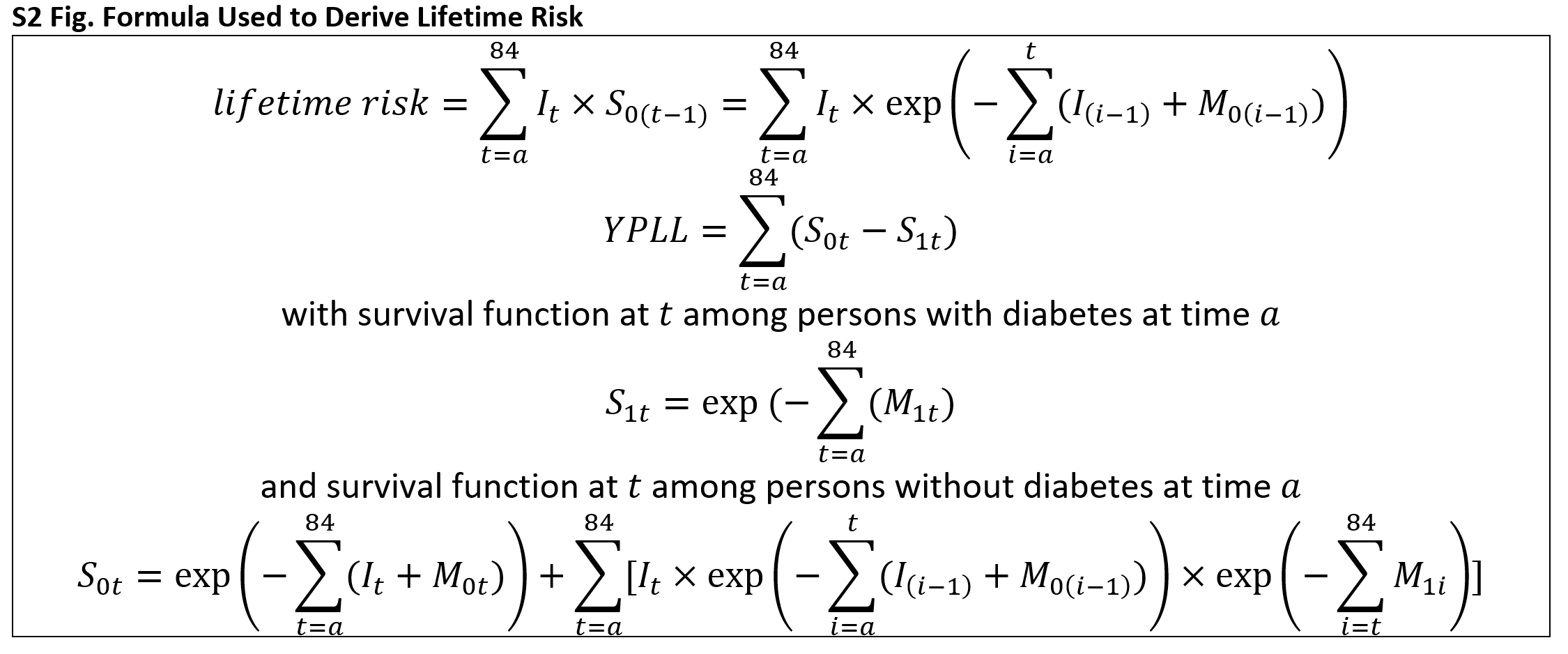

Supplement: S2 Fig — (TIF) [file pone.0268805.s008.tif]
